# Elevated Red Cell Distribution Width to Platelet Ratio Is Associated With Poor Prognosis in Patients With Spontaneous, Deep-Seated Intracerebral Hemorrhage

**DOI:** 10.3389/fneur.2021.751510

**Published:** 2021-11-15

**Authors:** Felix Lehmann, Lorena M. Schenk, Joshua D. Bernstock, Christian Bode, Valeri Borger, Florian A. Gessler, Erdem Güresir, Motaz Hamed, Anna-Laura Potthoff, Christian Putensen, Matthias Schneider, Julian Zimmermann, Hartmut Vatter, Patrick Schuss, Alexis Hadjiathanasiou

**Affiliations:** ^1^Department of Anesthesiology and Intensive Care, University Hospital Bonn, Bonn, Germany; ^2^Department of Neurosurgery, University Hospital Bonn, Bonn, Germany; ^3^Department of Neurosurgery, Brigham and Women's Hospital and Harvard Medical School, Boston, MA, United States; ^4^Department of Neurosurgery, University Hospital Rostock, Rostock, Germany; ^5^Department of Neurology, University Hospital Bonn, Bonn, Germany

**Keywords:** spontaneous intracerebral hemorrhage, red blood cell distribution width, platelets, morbidity/mortality, inflammation

## Abstract

**Object:** Inflammatory response is an important determinant of subsequent brain injury after deep-seated intracerebral hemorrhage (ICH). The ratio of red blood cell (RBC) distribution width to platelet count (RPR) has been established as a new index to reflect the severity of inflammation. To the best of our knowledge, no association between RPR and prognosis after spontaneous ICH has yet been reported.

**Methods:** In all patients with deep-seated ICH treated at our Neurovascular Center from 2014 to 2020, initial laboratory values were obtained to determine RPR in addition to patient characteristics and known risk factors. Subsequent multivariate analysis was performed to identify independent risk factors for 90-day mortality after deep-seated ICH.

**Results:** Hundred and two patients with deep-seated ICH were identified and further analyzed. Patients with an initial RPR < 0.06 exhibited significantly lower mortality rate after 90 days than those with an initial RPR ≥ 0.06 (27 vs. 57%; *p* = 0.003). Multivariate analysis identified “ICH score ≥ 3” (*p* = 0.001), “anemia on admission” (*p* = 0.01), and “elevated RPR ≥ 0.06” (*p* = 0.03) as independent predictors of 90-day mortality.

**Conclusions:** The present study constitutes the first attempt to demonstrate that the ratio of RBC distribution width to platelets—as an independent inflammatory marker—might serve for prognostic assessment in deep-seated ICH.

## Introduction

Patients that suffer from spontaneous intracerebral hemorrhage (ICH) often display significant morbidity/mortality due to the extent and/or the location of the bleed (1). These devastating injuries occur as a result of primary [i.e., mass effect(s)] and/or secondary (i.e., oxidative stress, inflammation, etc.) injury caused by the hemorrhage (2). Of note, deep-seated ICH appears to be distinct from lobar ICH, implying different etiologies and ultimately different clinical outcomes (3).

Given the varying degrees and locations of ICH, reliable prognostic aids supporting early clinical decision-making are essential. Accordingly, herein we assessed ICH scores, and a myriad of lab values in effort to develop a tool capable of providing better clinical guidance for neurointensivists, neurosurgeons, ICH patients, and their families (4–7).

Of note, the inflammatory response referenced above is exacerbated via the intracerebral extravasation of blood products (8, 9). Intracerebral hemorrhage-induced inflammation precedes disruption of the blood-brain barrier (BBB) and in patients with spontaneous ICH, concurrent alterations of immune profiles within peripheral blood have been observed (10). Such alterations in peripheral blood have been the target of numerous efforts to establish markers for early ICH prognostication (11–13).

Red cell distribution width (RDW), an indicator of size variability among circulating red blood cells (RBCs), is routinely reported as part of the complete blood count (CBC) and is typically used to identify the etiology of anemia (14). More recently, RDW has gained considerable attention as an inflammatory marker and as a predictive metric for cardiac as well as infectious diseases (15–17). In addition, there is evidence that RDW is a significant prognostic factor in several malignancies (18, 19). A new index, the ratio of RDW to platelet count (RPR), has been reported to reflect the severity of inflammation and has previously been utilized for prognostic prediction in breast cancer or fibrosis in chronic hepatitis (20, 21).

To the best of our knowledge, no association between RPR and prognosis after spontaneous ICH has been reported. Therefore, the aim of the present work was to include the RPR as a simple, readily available index for early prognostic assessment in patients with spontaneous deep-seated ICH to facilitate intensive care decision making.

## Materials and Methods

This retrospective study includes 102 patients with spontaneous deep-seated ICH who were referred to our Neurovascular Center for further management between 2014 and July 2020. The institutional ethics review board approved this retrospective study (no. 383/20). All procedures used in this study were performed in accordance with the principles of the Declaration of Helsinki and its subsequent amendments. To obtain a more homogeneous patient cohort, patients with traumatic or other bleeding sources (e.g., aneurysm, arteriovenous malformation, tumor) and those with a lobar ICH location were excluded from further analysis. All patients received at minimum a conservative treatment, including blood pressure control, according to current guidelines. Information on patient characteristics, ICH localization, ICH extent, ICH score (4), and intensive care and laboratory values were collected and analyzed in a computerized database. Furthermore, patients were separated into two groups based on the ICH score. Given the experience from previous studies on the clinical significance of the ICH score pertaining to mortality, the groups were divided into patients with an initial ICH score <3 and an ICH score ≥3 (4, 22). Blood samples were obtained from peripheral venous blood as part of routine management at the time of hospital admission and thus before any therapeutic interventions. Red cell distribution width and platelet count were routinely obtained. The ratio of RDW to platelets was calculated accordingly at follow-up. Regarding further inflammatory markers, patients were divided into two groups each based on their C-reactive protein (CRP; CRP ≤ 3 mg/l vs. CRP > 3 mg/l), procalcitonin value (PCT; PCT ≤ 0.5 μg/l vs. PCT > 0.05 μg/l), and peripheral white blood count (WBC; WBC ≤ 12g/l vs. WBC > 12g/l) at the time of hospital admission (6). Anemia assessed in the admission laboratory was defined according to the World Health Organization (WHO) definitions [hemoglobin (Hb) <12 g/dl for women, Hb <13 g/dl for men] (23).

Mortality at 90 days after the bleeding event was used as the primary end point. Thus, to avoid confounding bias of the results attributable to cases with devastating extent of hemorrhage and/or patient wishes of waiving life-sustaining measures, only patients hospitalized for >3 days were included. Data analysis was accomplished using the SPSS computer software package (version 25, IBM Corp., Armonk, NY, USA). The Mann-Whitney U-test was chosen to compare continuous variables because the data were mostly not normally distributed. Categorical variables were analyzed in contingency tables using Fisher's exact test. Results with *p* < 0.05 were deemed statistically significant. To assess the discrimination ability of the ICH score and the RPR in predicting the probability of a case fatality within 90 days, receiver operating characteristic (ROC) curves were constructed for both values within the patient population studied, and the areas under the curves (AUC) were calculated. The optimal cut-off value for each curve was determined for sensitivity and specificity, both being equally important. In addition, to ascertain independent predictors of 90-day mortality in patients with deep-seated ICH, multivariate analysis was performed using binary logistic regression.

## Results

### Patient Characteristics

Between 2014 and July 2020, 102 patients with deep-seated ICH were referred to the corresponding authors' neurovascular specialty center for management/treatment of ICH. The median age of the patients was 66 years [interquartile range (IQR) 57–76 years]. At the time of admission, 64 patients (63%) presented with a Glasgow Coma Score (GCS) <13, 63 patients (62%) suffered from additional intraventricular hemorrhage (IVH), 53 patients (52%) had deep-seated ICH with hematoma volume ≥30 ml. The 90-day mortality rate was 42% (43/102 patients). Further details on patient characteristics, focusing on availability of data at the time of admission and stratified by 90-day mortality into two groups for survivors and non-survivors, are given in Table 1.

**Table 1:** Patient characteristics.

	**Survivors**	**Non-survivors**	
	**(*n* = 59)**	**(*n* = 43)**	
Median age (years, IQR)	63 (53–72)	68 (59–81)	*p* = 0.03
Female sex	21 (36%)	16 (37%)	*p* = 1.0
ICH score > 3	4 (7%)	16 (37%)	*p* = 0.0002, OR 8.1, 95% CI 2.5–26.7
Initial SBP (mmHg)			*p* = 0.7
Mild (<180)	36 (61%)	27 (63%)	
Moderate (180–219)	17 (29%)	10 (23%)	
Severe (≥220)	6 (10%)	6 (14%)	
Admission CRP (median, IQR; mg/l)	3.1 (1.2–9.2)	3.7 (1.5–10.4)	*p* = 0.6
Admission PCT (median, IQR; μg/l)	0.06 (0.04–0.12)	0.09 (0.05–0.27)	*p* = 0.01

### Influence of Anemia and Routine Inflammatory Markers on Mortality

Patients with anemia at the time of admission had a significant difference in 90-day mortality compared to patients without initial anemia (69 vs. 32%; *p* = 0.001, OR 4.8, 95% CI 1.9–12.2). No significant difference in mortality was observed in patients with an initial WBC of >12 g/l compared to patients with a baseline WBC ≤ 12 g/l (47 vs. 39%; *p* = 0.5). Ninety-day mortality following ICH was not significant in patients with a baseline CRP ≤ 3 mg/l compared with patients with an initial CRP > 3 mg/l (38 vs. 45%; *p* = 0.6). Patients with deep-seated ICH and an initial PCT ≤ 0.5 μg/l exhibited significantly lower mortality at 90 days compared to patients with an initial PCT > 0.5 μg/l (39 vs. 78%; *p* = 0.03, OR 5.5, 95% CI 1.1–28.2).

### Influence of RPR on Mortality

Neither the median admission laboratory value for RDW nor that for platelet count demonstrated a significant difference between survivors and non-survivors with deep-seated ICH (*p* = 0.2 and *p* = 0.07, respectively). The median RPR for the entire cohort was 0.062 (IQR 0.050–0.078). The optimal cut-off value in terms of RPR was determined to be 0.06 with an AUC of 0.63 (95% CI 0.517–0.739; *p* = 0.027) and a sensitivity of 70% and a specificity of 61%. Table 2 illustrates the distribution of known and established risk factors for poor outcome in patients with deep-seated ICH. With the exception of age, none of the known prognostic factors are statistically different between the two groups. Patients with an RPR < 0.06 were distinguished by significantly lower case-fatality at 90 days compared to patients with a baseline RPR ≥ 0.06 (27 vs. 57%; *p* = 0.003, OR 3.6, 95% 1.6–8.3).

**Table 2:** Distribution of known prognostic factors in patients with deep-seated ICH.

	**RPR <0.06**	**RPR ≥ 0.06**	
	**(*n* = 49)**	**(*n* = 53)**	
ICH score > 3	8 (16%)	12 (23%)	*p* = 0.5
Age ≥ 80 years	4 (8%)	13 (25%)	*p* = 0.03, OR 3.7, 95% CI 1.1–12.1
Baseline GCS <13	30 (61%)	34 (64%)	*p* = 0.8
Baseline ICH volume ≥ 30 ml	22 (45%)	31 (59%)	*p* = 0.2
Initial IVH	27 (55%)	36 (68%)	*p* = 0.2
CRRT	2 (4%)	8 (15%)	*p* = 0.1
PMV (>7 days)	21 (43%)	25 (47%)	*p* = 0.7
90 day mortality	13 (27%)	30 (57%)	*p* = 0.003, OR 3.6, 95% 1.6–8.3

### Multivariate Analysis

**Figure 1:**
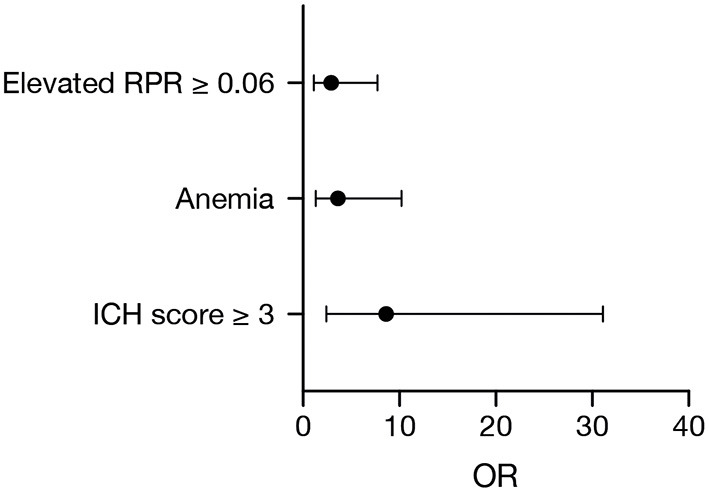
Forest plot of significant and independent predictors of 90-day mortality identified by multivariate logistic regression analysis.

Multivariate logistic regression analysis was performed to identify independent initial determinable predictors of 90-day mortality in patients with deep-seated ICH and to clarify the influence of the RPR in this regard. Multivariate analysis identified “ICH score ≥ 3” (*p* = 0.001, OR 8.6, 95% CI 2.4–31.1), “anemia on admission” (*p* = 0.01, OR 3.6, 95% CI 1.3–10.2), and “elevated RPR ≥ 0.06” (*p* = 0.03, OR 2.9, 95% CI 1.1–7.7) as significant and independent predictors of 90-day mortality (Nagelkerke's *R*^2^ = 0.35, Figure 1).

## Discussion

The present study evaluated a potential link between RPR and 90-day mortality after deep-seated ICH. The results suggest that patients with an elevated RPR in the admission laboratory are more likely to decease within 90 days after the bleeding.

RPR is calculated from the ratio of RBC distribution width to platelet count. As a potential indicator of inflammatory processes, RPR has already been established as a prognostic factor in numerous other diseases. In patients with ICH, to the best of our knowledge, there have been no such association studies yet. In the context of the initial bleeding, but also in the course of the alteration/depletion processes in the area of the hemorrhage, a number of inflammatory reactions have been detected in ICH (24, 25). Not only via the initial extravasation of cytokines, but also through blood breakdown products, inflammatory reactions may very well have a direct impact on the pathophysiological and clinical course, e.g., on the development of perilesional edema (26). Thus, early and readily available inflammatory markers may provide an important insight into the inflammatory events early in the course of management/treatment of patients with ICH and thereby offer a reflection of the inflammation-triggered brain injury in the setting of ICH (27, 28).

RPR is derived in part from RDW. Red cell distribution width measures the size distribution of RBCs and indicates in case of strongly elevated values the presence of different types of anemia. Anemia or low hemoglobin have already been identified as influencing factors on outcome but also on hematoma extent after spontaneous ICH (29–31). In the present work, anemia is also associated with increased mortality, and yet the RPR extends beyond a mere determination of hemoglobin by including a platelet count. RPR has been found to be a prognostic marker in various immunological diseases and is also considered to reflect the severity of the inflammatory event by attributing some influence to platelet count (20, 21). In the inflammatory process, in addition to sepsis-related anemic events, inflammatory cytokines may inhibit the maturation of RBCs and thus alter the RDW (32) whereas platelets are known to be placed on the intersection of an immune response and coagulation in infectious diseases (33). Thus, a marker as RPR, which takes into account both potential reflections of the inflammatory process, may be more accurate than non-specific markers such as CRP (34, 35). Also, in the anticipation of further complications in the course of treatment of ICH (e.g., renal replacement therapy), non-specific markers such as CRP have been reported to be less predictive than those that are considered more specific for inflammatory processes, such as PCT (6). Common to non-specific inflammatory markers may be a reflection of just another surrogate parameter as for example, in terms of a stress response of the body to intracranial hematoma volume or aspiration in the context of low GCS.

Nevertheless, when discussing the potential implications of this readily available laboratory marker, it is important to note that the focus of the present study was directed at patients with deep-seated ICH. One reason for this selection lies in the multiple etiologic possibilities in patients with lobar ICH. An underlying disease resulting in lobar ICH (e.g., cancer) seems to be more likely to affect initial laboratory parameters compared to deep-seated ICH (3).

The present study demonstrates the anticipated distribution of known and/or established risk factors for increased mortality after ICH (except for age) in both groups with decreased and increased RPR. This strengthens the suspicion that the RPR could be established as an independent prognostic factor. Despite the association of RPR with mortality after deep-seated ICH reported herein, further exploration of the underlying mechanisms and corroboration of our findings in other study populations is warranted.

## Limitations

In addition to retrospective data collection and analysis with the bias inherent to this study design, our study reveals other shortcomings that need to be carefully considered when interpreting our results. The sample size is comparably small due to a restriction to patients with a deep-seated ICH. This increases the homogeneity of the study population, but also raises the possibility of selection bias. Among these is the exclusion of patients with lobar ICH. However, this might also be considered a potential advantage, as this pre-selection might reduce the variety of underlying ICH etiologies and therefore their potential influence on initial laboratory values. In addition, the retrospective data analysis also precludes a dedicated analysis of potential additional determinants (such as a preexisting chronic inflammatory disease/acute aspiration pneumonia) throughout the complete dataset.

## Conclusion

This is the first study to provide evidence that RBC distribution width to platelet ratio might constitute an independent marker of inflammation associated with the prognostication of outcome in deep-seated ICH.

## Data Availability Statement

The original contributions presented in the study are included in the article/supplementary material, further inquiries can be directed to the corresponding author/s.

## Ethics Statement

The studies involving human participants were reviewed and approved by Local Ethics Committee at the University of Bonn. Written informed consent for participation was not required for this study in accordance with the national legislation and the institutional requirements.

## Author Contributions

FL, PS, and AH: conceptualization and supervision. FL, LS, PS, and AH: data curation. FL, PS, MS, and AH: formal analysis. FL, JB, FG, MS, PS, and AH: writing—original draft preparation. FL, LS, JB, CB, VB, FG, EG, MH, A-LP, CP, MS, JZ, HV, PS, and AH: writing—review and editing. A-LP and MS: visualization. All authors contributed to the article and approved the submitted version.

## Conflict of Interest

The authors declare that the research was conducted in the absence of any commercial or financial relationships that could be construed as a potential conflict of interest.

## Publisher's Note

All claims expressed in this article are solely those of the authors and do not necessarily represent those of their affiliated organizations, or those of the publisher, the editors and the reviewers. Any product that may be evaluated in this article, or claim that may be made by its manufacturer, is not guaranteed or endorsed by the publisher.
